# Prospective Surveillance of Healthcare-Associated Infections in Residents in Four Long-Term Care Facilities in Graz, Austria

**DOI:** 10.3390/antibiotics10050544

**Published:** 2021-05-07

**Authors:** Elisabeth König, Mara Medwed, Christian Pux, Michael Uhlmann, Walter Schippinger, Robert Krause, Ines Zollner-Schwetz

**Affiliations:** 1 Section of Infectious Diseases and Tropical Medicine, Department of Internal Medicine, Medical University of Graz, 8036 Graz, Austria; elisabeth.ullrich@medunigraz.at (E.K.); mara.medwed@stud.medunigraz.at (M.M.); robert.krause@medunigraz.at (R.K.); 2 Geriatric Health Centers of the City of Graz, 8020 Graz, Austria; christian.pux@stadt.graz.at (C.P.); michael.uhlmann@stadt.graz.at (M.U.); walter.schippinger@stadt.graz.at (W.S.)

**Keywords:** health-care associated infections, nosocomial infections, long term care facility, nursing homes

## Abstract

Healthcare-associated infections (HCAI) are a common cause for residents’ mortality and morbidity associated with a significant socio-economic burden. Data on HCAIs in Austrian long-term care facilities are scare. Therefore, we evaluated the incidence rate of HCAIs per 1000 resident days in four LTC facilities in Graz, Austria, characterized the spectrum of HCAIs and the use of antimicrobial substances. We conducted a prospective surveillance study from 1 January to 31 December 2018 in four LTCFs of the Geriatric Health Centre of the City of Graz (total of 388 beds). Nursing staff collected data on HCAIs once a week using an electronic reporting system. During the 12-month surveillance period, 252 infections of 165 residents were recorded. The overall incidence rate of HCAIs was 2.1 per 1000 resident days. Urinary tract infections were the most commonly recorded HCAIs (49%, 124/252, 1.03 per 1000 resident days), followed by skin and soft tissue infections and respiratory tract infections. Beta-lactams (ATC class J01C) were prescribed most frequently (63/212), followed by fluoroquinolones (J01M; 54/212). In conclusion, the overall incidence rate for HCAIs was relatively low at 2.1 per 1000 resident days. Our real-life data can serve as a basis for future antimicrobial stewardship and infection prevention interventions.

## 1. Introduction

As life expectancy is increasing continuously in the industrialized world, long-term care facilities for the elderly play an essential role in contemporary healthcare systems due to an ageing population [1]. Population projections calculated that by 2100 there will fewer than two persons of working age for each elderly person aged 65 in the European Union [2].

Healthcare-associated infections are common in the vulnerable population in long-term care facilities [3,4,5,6,7,8]. Healthcare-associated infections are those infections that patients acquire while receiving healthcare in any healthcare facility, including long-term care facilities [9]. Reduced immunological competence, as well as often multiple comorbidities, chronic illnesses and implanted foreign materials, such as prosthetic joints and catheters, contribute to the vulnerability to nosocomial infections [10,11,12]. Factors contributing to the spread of healthcare-associated infections in long-term care facilities include difficulties in diagnosing infections because of atypical clinical presentation and cognitive impairment of residents [13,14,15,16]. The timely and accurate diagnosis of infections in the elderly requires expertise in this area [17,18]. Limited access to laboratories and radiology may also play a role by hampering speedy and adequate diagnostic workups [19]. In addition, the knowledge on how to prevent healthcare-associated infections and the importance attributed to infection prevention measures are often unsatisfactory in healthcare workers [20].

Available data from the United States indicate that infections are an increasing problem for long-term care facilities and nursing homes [21]. In addition, infections at long-term care facilities are a common cause for residents’ mortality and morbidity associated with a significant socio-economic burden [22,23]. According to a review by Lapane et al., the success of reducing healthcare associated infections in nursing homes has been limited [24].

To quantify the magnitude of healthcare-associated infections in long-term care facilities at a European level, the European Centre for Disease Control (ECDC) provided funding for the HALT project (healthcare-associated infections in long-term care facilities). This point-prevalence study evaluated the prevalence of healthcare-associated infections and explored antimicrobial use in long-term care facilities throughout Europe in 2010 [25]. The survey was repeated in 2013 and in 2016–2017 [26,27]. In 2016, the prevalence of residents with at least one healthcare-associated infection was 3.1%, and ranged from 0.6% in Lithuania to 6.2% in Spain [27]. Although Austria was part of this international study, country data representativeness was poor because of a low number of participating long-term care facilities [27]. As a result of their studies the ECDC recommended “to continue monitoring of healthcare-associated infections and antimicrobial use” in long-term care facilities.

Antibiotics are one of the most frequently prescribed medications in long-term care facilities [28]. Studies have shown that 30–40% of antibiotic courses prescribed in nursing homes were unnecessary [29,30]. Thus far, the data on healthcare-associated infections, and especially use of antimicrobial agents for Austrian long-term care facilities are very limited. Therefore, the aims of our study were: (1) to evaluate the incidence rate of healthcare-associated infections per 1000 resident days in four long-term care facilities in Graz, Austria; (2) to characterize the spectrum of healthcare-associated infections; and (3) to study the use of antimicrobial substances.

## 2. Results

During the 12-month surveillance period, 252 infections of 165 residents (120/165 female) were recorded (119,957 resident days). The mean age of residents was 85.6 ± 9.1 years (median = 87 years, range 57–102 years). Fifty-seven percent (94/165) of residents were older than 85 years. The overall incidence rate of healthcare-associated infections was 2.1 per 1000 resident days. The incidence rate varied by long-term care facility and ranged from 1.7/1000 resident to 2.8/1000 resident days. Only 4/252 (1.5%) healthcare-associated infections were acquired outside the LTCFs.

### 2.1. Types of Infections

Urinary tract infections (UTI) were the most commonly recorded healthcare-associated infections (49%, 124/252, 1.03 per 1000 resident days). Of those, 9.6% (12/124) were device-associated. Urinary cultures were performed in 8/124 (6.5%) of UTI episodes and all were positive. The remaining 116 episodes (93.5%) were “probable” UTIs according to the HALT classification because no urinary cultures were performed and 30/124 (24.2%) were recurrent infections.

Seventy-four episodes of skin soft tissue and mucosal infections were recorded (29%, 74/252, 0.62 per 1000 resident days). This category included infections of eyes, ears and teeth as well (Table 1). Two cases of herpes zoster were detected. Dermatomycosis occurred in 32/74 cases.

Forty-three episodes of lower RTIs were documented (43/252, 17%, 0.36 per 1000 resident days). Among these was one case of proven influenza. Two thirds of lower RTIs (25/43) occurred in the winter months (November–April).

Only five episodes of gastroenteritis were detected in four patients (5/252, 1.9%, 0.04 per 1000 resident days). One patient experienced two episodes of a *Clostridioides difficile* infection. Six episodes of unexplained febrile illness occurred (6/252, 2.3%, 0.05 per 1000 resident days) during the surveillance period.

### 2.2. Antimicrobial Therapy

**Figure 1:**
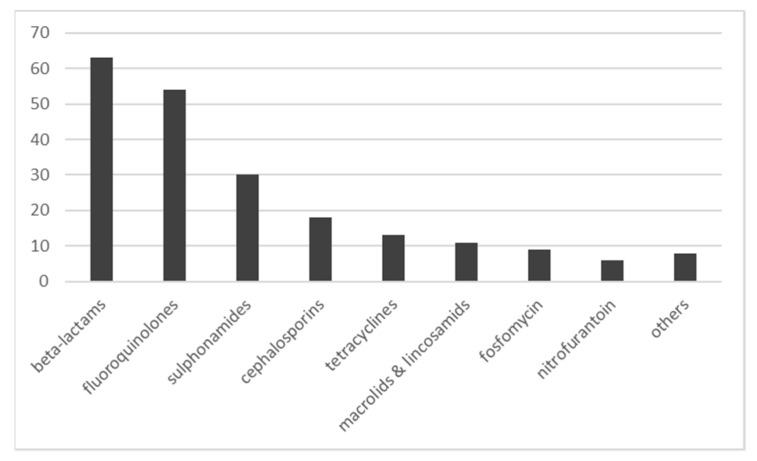
Systemic antimicrobial substances for the treatment of 212 health care associated infections.

During the survey period, 212/ 252 (84%) infections were treated with at least one antimicrobial agent for systemic use (Anatomical Therapeutic Chemical (ATC) class J01), resulting in an incidence rate of 1.77 per 1000 resident days. Beta-lactams (ATC class J01C) were prescribed most frequently (63/212; 29.7%) followed by fluoroquinolones (J01M; 54/212, 25.5%), sulfonamides (J01E; 30/212, 14.1%) and cephalosporins (J01D; 18/212, 8.5%), Figure 1.

**Figure 2:**
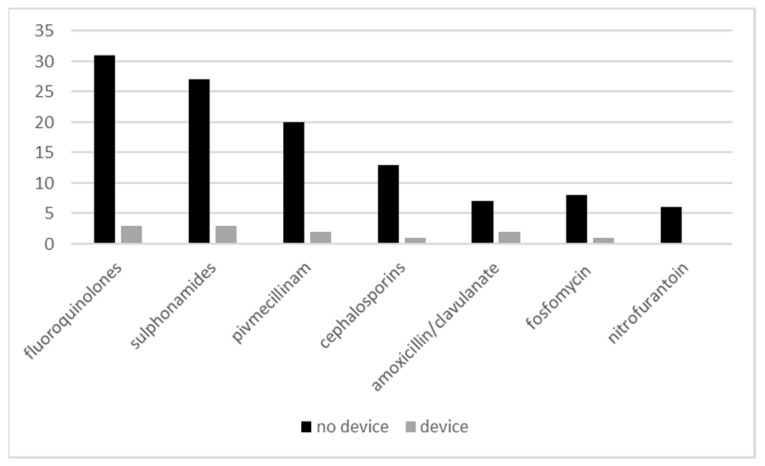
Antimicrobial substances used to treat 112 non-device associated and 12 device-associated urinary tract infections.

Six UTI episodes were treated with more than one antimicrobial substance. Only the second, presumably successful treatment was included in the analysis. Two UTI episodes in one resident were treated with herbal substances followed by ciprofloxacin. Fluoroquinolones (ATC class J01M) were used most frequently to treat UTI episodes (34/124, 27.4%) followed by sulfonamides (J01E; 30/124, 24.2%) and pivmecillinam (J01CA08; 22/124, 17.7%), Figure 2. Mean duration of antimicrobial therapy was 7.2 days.

For the treatment of skin and mucosal infections beta-lactams (J01C) were used most frequently (17/74, 23%), followed by fluoroquinolones (J01M; 8/74, 10.8%) and clindamycin (J01FF01; 6/74, 8%). Mean duration of antimicrobial therapy was 11.4 days. Oral acyclovir (J05AB01) was prescribed for both patients with Herpes zoster for 7 days. Topical agents were used in 40/74 (54%) of skin and mucosal infections mainly antifungal substances (31/40, 77.5%).

Amoxicillin/clavulanate (J01CR02) was used most commonly to treat lower RTIs (15/43, 34.8%) followed by fluoroquinolones (J01M; 10/43, 23.2%) and doxycycline (J01AA02; 11/43, 25.6%). Mean duration of antimicrobial therapy was 7.6 days. One patient with influenza was treated with oseltamivir (J05AH02) for 7 days.

One patient suffered from a recurrent *C. difficile* infection, the first of which was treated with rifaximin (A07AA11), the second (three weeks later) with oral vancomycin (A07AA09). The other patient received metronidazole (J01XD01) (mean duration of *C. difficile* therapies 8.3 days).

Six cases of unexplained febrile illness occurred. Two patients were treated with amoxicillin/clavulanate, two patients with fluoroquinolones, one with clarithromycin and one with doxycycline.

## 3. Discussion

In our study, the overall incidence rate of healthcare-associated infections was 2.1 per 1000 resident days which is lower than previously described in studies from Germany (range 5 to 6 per 1000 resident days) and the United States (range 3.6 to 7.1 infections/1000 patient-days) [31,32,33,34,35]. Most of these investigations were undertaken between 2000 and 2006 [31,34,35] or even earlier [33]. A Dutch study showed a trend for a decrease in healthcare-associated infections in long-term care facilities from 2007 to 2017 [36]. It is possible that overall improved standards of care in long-term care facilities have led to a decrease in healthcare-associated infections that would be detectable also in other areas of the world. Differences in the definitions of healthcare-associated infections could also be responsible for variable results. In our study, local nursing staff recorded healthcare-associated infections only when the patient’s general practitioner diagnosed an infection. Therefore, upper respiratory tract infections such as common cold or pharyngitis that were treated by residents and nurses without consulting a general practitioner were not recorded. This may have led to an underestimation of healthcare-associated infections overall. However, upper respiratory infections only accounted for about 30 to 40% of RTIs in two studies [31,32]. If we increase the number of RTIs by 40% to make up for the estimated missing upper RTIs, the overall incidence rate of healthcare-associated infections would increase only marginally to 2.3/1000 resident days and would still be lower than previously described [31,32].

The most common healthcare-associated infections in our study were UTIs, as was found by other authors including Suetens et al. presenting data on two European point prevalence surveys [27,32,36]. Microbiological testing was only undertaken in 6.5% of UTIs. This is in contrast to results from an Italian study where urine cultures were performed in 46% of UTIs [19]. A possible explanation could be that a centralized medical service is not available in the long-term care facilities we studied, making it more difficult to implement uniform standards of diagnostic steps.

The overall incidence rate of healthcare-associated infections varied by nursing home (0.47–1.78 per 1000 resident days) as has been described before [10]. Heterogeneous populations of residents or differences in approaches to infection control standards could explain these differences. In LTC facility 4 an increased rate of UTIs was detected in the first half 2018. Therefore, the local infection control team initiated an intervention including review of existing guidelines, especially on handling of indwelling catheters, audits and on-site trainings. We excluded nursing home 4 from our analysis in the second half of 2018 to avoid bias.

During the surveillance period, beta-lactams (ATC class J01C) were the most frequently prescribed systemic antimicrobial substances (30%). This is in line with data from the Austrian national report on antimicrobial resistance and antimicrobial use [37]. In this report, antimicrobial use is documented not exclusively for long-term care facilities but rather for all patients in acute care and outpatients [37]. In 2017, betalactams (J01C) accounted for 54% of the overall consumption of systemic antimicrobial substances in Austria [37]. In our study, fluoroquinolones (J01M) constituted 25% of all prescriptions compared to 5 % for macrolides and lincosamids (J01F). These findings are in contrast to data from AURES where ATC class J01F is the second most common group used in the general population outside hospitals followed by fluoroquinolones [37]. A recent international study on antimicrobial use in European long-term care facilities described similar findings for Austria as are reported here, i.e., betalactams were most frequently prescribed followed by fluoroquinolones, underlining the validity of our data [1].

Our study has limitations. Nursing staff recorded healthcare-associated infections only when the patient’s general practitioner diagnosed an infection. This has most likely led to an underestimation of RTIs as only lower RTIs were treated by a physician and hence recorded. In addition, there may have variations in the diagnosis of infections by general practitioners between patients and long-term care facilities. Taken together, this may reduce the generalizability of our data. However, our real-life data can serve as a basis for future antimicrobial stewardship and infection prevention interventions.

## 4. Materials and Methods

### 4.1. Setting and Study Design

We conducted a prospective surveillance study from 1 January to 31 December 2018 in the Geriatric Health Centre of the City of Graz. It is a local institution comprising 4 long-term care facilities situated all around the city of Graz. In Austria, there were 817 long-term care facilities in 2017 [27]. A total of 24 nursing homes is located in the city of Graz [38]. The total number of beds was 388. The LTC facilities provide 312 single rooms with personal toilets and 76 double rooms. Three long-term care facilities are structured in smaller units of 13 to 15 patients sharing a living room. One facility is structured into units for 18 to 27 residents. The long-term care facilities studied offer assistance with activities of daily living and skilled nursing care (e.g., use of urinary catheters or enteral feeding tubes) as needed. They do not deliver on-site medical care and do not have a centralized medical service. Instead, residents keep their own general practitioner after moving to the long-term care facilities. Seven to ten different general practitioners per long-term care facility deliver medical care at the institution during weekly scheduled visits and, if needed, on demand visits.

### 4.2. Data Collection and Definitions

Local nursing staff collected data on healthcare-associated infections once a week using an electronic reporting system. Collected variables included age, gender, type of infection, causative agent (if known), type and dose of antimicrobial therapy, length of therapy. In addition, it was documented whether the infection was device associated (e.g., associated with an indwelling urinary catheter) and whether it was acquired at the long-term care facility or elsewhere (e.g., during a hospital stay). Definitions of healthcare-associated infections were based on the ECDC HALT project [38]. However, a healthcare-associated infection was recorded only when the patient’s general practitioner was contacted by local nursing staff and diagnosed the infection. The following entities were documented: urinary tract infections (UTI), lower respiratory tract infections (RTI), skin and mucosal infections (including infections of the eyes, ears and teeth), gastrointestinal infections and unexplained febrile illness. A healthcare-associated infection was defined as a new infection if ≥7 days had passed between the end of the previous episode and the start of a new episode. Healthcare-associated infections were defined as a recurrence if the same healthcare-associated infection in the same patient occurred within 12 weeks.

In long-term care facility D an increased rate of UTIs was detected in the first half 2018. Therefore, the local infection control team initiated an intervention including review of existing guidelines especially, on handling of indwelling catheters, audits and on-site trainings. We therefore excluded data from long-term care facility D from July to December 2018 from this analysis. In long-term care facility C the rate of UTIs was also elevated. However, limited ICP resources did not allow immediate intervention in 2018. However, in 2019 measures were taken to address this issue in long-term care facility C.

### 4.3. Statistical Analysis

Quantitative variables were expressed as mean ± standard deviation. Categorical variables were expressed as percentages. The incidence rate per 1000 resident days of healthcare-associated infections in 4 long-term care facilities was calculated. The statistical software package SPSS 23 (Chicago, IL, USA) was used.

## 5. Conclusions

In conclusion, the overall incidence rate for healthcare-associated infections was 2.1 per 1000 resident days. To our knowledge, this is the first study on prospective surveillance of healthcare-associated infections in long-term care facilities in Austria.

## Figures and Tables

**Table 1:** Number of healthcare-associated infections and incidence rates during a 12-month surveillance period in four long-term care facilities.

Infection	Number of Infections (%)	Average Rate per 1000 Resident Days	Rate per 1000 Resident Days Unit A	Rate per 1000 Resident Days Unit B	Rate per 1000 Resident Days Unit C	Rate per 1000 Resident Days Unit D
Urinary tract infections	124	1.03	0.47	0.78	1.54	1.78
Skin, soft tissue and mucosal Wound infection Dermatomycosis Herpes zoster Other skin infections Eye and ear Teeth	74 26 32 2 8 3 3	0.62 0.22 0.27 0.02 0.07 0.03 0.03	0.88	0.62	0.38	0.53
Lower respiratory tract infections Influenza	43 1	0.36 0.01	0.56	0.22	0.25	0.47
Gastroenteritis *Clostridioides* difficile	5 3	0.04 0.03	0.12	0	0	0.06
Unexplained febrile illness	6	0.05	0.06	0.05	0.06	0
Total	252	2.1				

## Data Availability

The data presented in this study are available on request from the corresponding author. The data are not publicly available due to privacy concerns.
